# Precision Immunosuppression in Heart Transplantation: Biomarker-Guided Therapy Comes of Age

**DOI:** 10.1007/s11897-026-00784-7

**Published:** 2026-10-14

**Authors:** Karthik Sreedhara, Palak Shah

**Affiliations:** 1 Division of Cardiology, George Washington University School of Medicine and Health Sciences, Washington, DC 20037 USA; 2 Division of Cardiology, Department of Medicine, Northwestern University Feinberg School of Medicine, 676 N St Clair Street, Chicago, IL 60611 USA

**Keywords:** Heart transplantation, Immunosuppression, Precision medicine, Biomarkers, Donor derived cell-free DNA, Gene expression profiling

## Abstract

**Purpose of Review:**

Heart transplantation is the definitive treatment for end-stage heart failure. While the number of heart transplant performed yearly in the United States has nearly doubled in the last decade, median survival has stagnated, largely due to the complications posed by lifelong immunosuppression. A shift towards precision medicine is needed to achieve the optimal balance between risk of allograft rejection and drug-related toxicities to improve post-transplant outcomes.

**Recent Findings:**

Novel molecular based diagnostic tools and biomarkers are being investigated, and hold promise in evaluating graft health and assessing immune state.

**Summary:**

The use of circulating biomarkers and molecular diagnostics such as human and viral cell-free DNA, tissue-based gene expression profiling, and circulating microRNAs, will allow for more comprehensive assessment of post-transplant immune activity and rejection risk to personalize patient care.

## Introduction

Heart transplantation remains the definitive therapy for advanced heart failure, offering the most favorable survival benefit and risk profile long term [1]. During the initial explosion of interest in human heart transplantation in the 1960s, development of the surgical technique was the primary focus, but the procedure quickly lost favor by the 1970s in most centers due to excessive mortality rates attributed to acute organ rejection. It was not until the discovery and approval of cyclosporine in the 1980s that solid organ transplant and heart transplantation returned as a viable treatment for end-stage heart failure. From 1990 to 2013, the annual volume of heart transplants stagnated at ~ 4500 a year [2]. In the past decade there has been a rejuvenation of the field and exponential growth in transplant volumes with use of direct-acting antivirals to permit hepatitis C positive donation, enhanced organ preservation techniques, and the use of deceased after circulatory death (DCD) donors either with normothermic regional perfusion (NRP), or through normothermic “beating heart” procurement, hypothermic machine perfusion of a non-beating heart and recently rapid recovery with extended ultra-oxygenated preservation [3]. However, overall survival of patients after heart transplant remains largely unchanged, with median survival post-transplant of about 13 years [4]. In the contemporary era, the one-year risk of rejection approximates 20% [5], and the prevalence of cardiac allograft vasculopathy (CAV) is 25% at 5-years [6].

Most immunosuppression regimens consist of selective use of induction therapy based on pre-transplant risk factors (e.g. age, sex, race, sensitization status and renal function) and then long-term maintenance therapy with a second-generation calcineurin inhibitor (CNI), typically tacrolimus, an anti-metabolite, typically mycophenolate mofetil, and corticosteroids [7].

However, patients are at risk of developing complications of chronic immunosuppression. Though rates of infection have dropped with routine use of antimicrobial prophylaxis, it remains a significant cause of morbidity and mortality while on chronic immunosuppression. Particularly during the first year, infection is a leading cause of death and account for ~ 25% of deaths in heart transplant recipients [6]. Bacterial infections are the most common cause of infection overall, with rates of CMV infection rising after 6 months post-transplant [8]. The incidence of kidney disease is high in heart transplant recipients, with 51% of patients developing renal dysfunction at 5 years and 20% incidence of end-stage renal disease at 10 years [9, 10]. Chronic CNI induced vasoconstriction is known to contribute to nephrotoxicity, especially in the background of hypertensive, diabetic, and ischemic nephropathy [11]. Lastly, malignancy is an increasingly important concern as heart transplant recipients age with improving early survival, with malignancy reported to be the cause of death in ~ 24% of patients greater than 5 years out from transplant [6]. More than 10% of adults heart transplant recipients will develop malignancy before 5 years with skin cancer being the most common likely due to combination of reduced immune activity, the carcinogenic effects of the immunosuppressive medications, and the amplification of oncogenic viruses [12].

To offset the complications of immunosuppression, there has been a growing interest in minimizing immunosuppressive dosing, to balance the risk of graft rejection due to undertreatment and risk of drug-related toxicities. However, the typical immunosuppression regimen at most transplant programs follows a one-size-fits-all approach, with adjustment based on clinical events. Therefore, advancing heart transplantation will need more tailored immunosuppressive strategies that are personalized to a patient’s specific needs. This will require diagnostic tools which allow for more precise risk stratification to guide therapeutic decision-making to hopefully improve long-term patient outcomes.

**Fig. 1:**
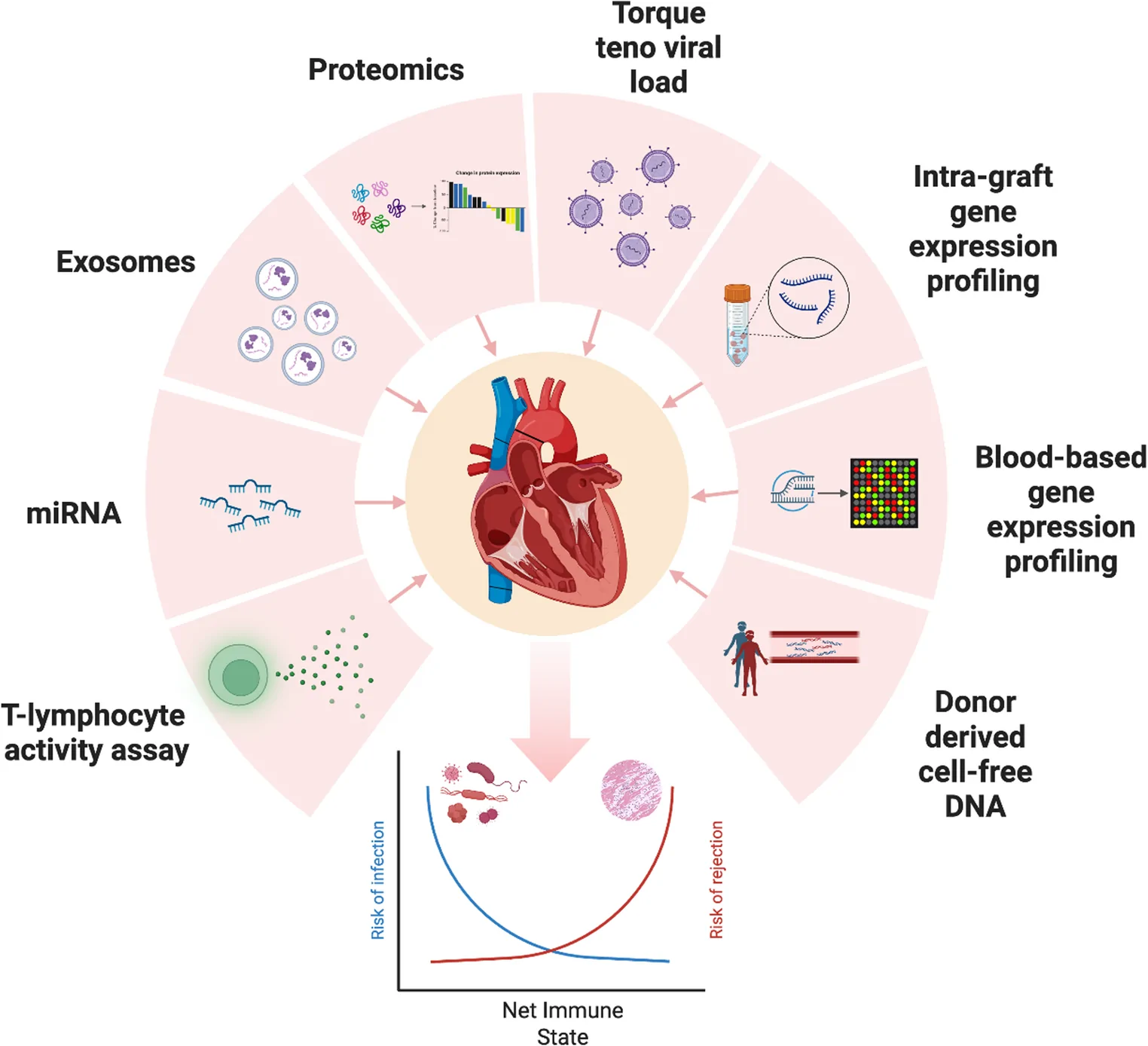
Using a multi-omic approach to help determine overall net immune state in order to reduce risk of complications of over- or under-immunosuppression. (Created with Biorender.com)

In this review we will highlight the role of novel biomarkers that are being used to not only identify and diagnose allograft rejection but can serve as biomarkers of immune function and net levels of immunosuppression (Fig. 1; Table 1). We review emerging strategies for immune monitoring which will pave the road towards personalized precision immunosuppression for heart transplant recipients.

**Table 1:** Summary of currently available and emerging molecular modalities for heart transplant

Technology	Description	Uses	Pros	Cons
GEP	Measures mRNA transcripts of 11 reporter genes and 9 normalization genes, from host circulating peripheral blood mononuclear cells, provides a rejection score	Non-invasive evaluation of ACR > 6 months post-transplant	High NPV for ACR	Not validated in AMR Cost
dd-cfDNA	Evaluation of single nucleotide polymorphisms (SNP) in circulating small DNA fragments (~ 150 base pairs). Quantified as ratio of donor to total serum cfDNA or absolute quantification of donor cfDNA	Assess for multiple mechanisms of graft injury and rejection	High NPV for both AMR and ACR, higher PPV than GEP can potentially differentiate between AMR and ACR	Cost
Intra-graft gene expression profiling	Intragraft mRNA transcripts from EMB samples	More accurately assess mechanism of graft injury after EMB, in AMR and ACR	Better correlates with degree of injury and disease activity than immuno-histology alone	Cost Requires repeat EMB Not widely available
Peripheral blood T-cell activation	Measures peripheral blood T-cell immunoreactivity	Evaluate immune status by assessment of T-cell activity	Potentially allows pharmacodynamic monitoring by measuring T-cell activation and proliferation	Not predictive of acute rejection or clinically significant infection, low level of evidence
microRNA**	Measures ~ 22 bp noncoding RNA molecules in EMB and circulating microRNA in serum	Assess for graft injury and rejection	Can provide precise molecular mechanism of injury by measuring specific groups of microRNAs which can be differentially dysregulated in ACR and AMR	Not clinically available
Soluble protein markers	Quantifying serum biomarkers such as NT-proBNP, CRP, high-sensitivity troponin T, IL-6	Assess for graft injury, ischemia, or evidence of acute inflammation	Cheap and widely available	Mixed results and weak association with graft injury
Proteomics/Exosome**	Protein expression profiling from serum and circulating extra-cellular vesicles	Assess for graft injury or rejection, assess immune status	Simultaneously analyze hundreds of proteins involved in tissue injury and inflammation to understand molecular mechanism of injury Can potentially differentiate between AMR and ACR	Not clinically available
Torque Teno Virus**	PCR quantification of circulating TTV	Assessment of net immune status	Can potentially predict risk of complications due to over-immunosuppression or risk of rejection due to under-immunosuppression	Limited studies in heart transplant population

** not clinically available

## Multiomic Biomarkers (Currently Available)

### Blood-Based Messenger RNA Gene Expression Profiling

In acute cellular rejection (ACR) alloantigen presentation by antigen-presenting cells triggers a CD4+/CD8 + T-cell response within the myocardium and activates an inflammatory cascade [13]. Gene expression profiling (GEP) measures 11 messenger RNA (mRNA) transcripts from the host’s circulating peripheral blood mononuclear cells, with specific gene expression involved in platelet and T-cell function, steroid responsiveness, and hematopoiesis, providing information on the recipient’s immune state [14]. The advantage of this technique is that the unique molecular signature expressed is based on the direct quantification of several leukocyte-derived transcripts. The transcripts are quantified and a GEP score is provided (0–40) that has a high negative predictive value (NPV = 99.6%) for ≥2R ACR [14]. With respect to immunosuppression optimization, several abstracts, but no definitive studies have suggested that a lower GEP score may suggest over-immunosuppression and a heightened risk of infection/malignancy. Prospective clinical studies assessing the association between lower GEP scores to complications of over-immunosuppression are needed.

### Donor-Derived Cell-Free DNA

Donor-derived cell-free DNA (dd-cfDNA) is a biomarker of allograft injury. Mechanistically, small fragments of double-stranded DNA are released into blood stream with normal donor cell apoptosis and turnover with an increase in levels during acute rejection. By quantifying differences in donor and recipient-specific single nucleotide polymorphisms (SNPs), the proportion of dd-cfDNA can be quantified [15]. Although the initial assays performed shotgun sequencing of both donor and recipient DNA, commercial assays rely on highly polymorphic SNPs to differentiate donor and recipient DNA and no donor DNA is required [16–18]. The benefit of dd-cfDNA over blood-based GEP is the ability to identify both ACR and AMR with a high sensitivity and a strong NPV. In addition, dd-cfDNA is often elevated months before histopathologic evidence of rejection on biopsy, suggesting it could potentially be a biomarker of immune activity [19]. Studies have also shown dd-cfDNA, in addition to being a marker of injury, may itself also serve as pathogen-associated molecular patterns (PAMPs) and damage-associated molecular patterns (DAMPs), leading to further activation of multiple immune signaling pathways [20].

Many studies evaluating the performance of dd-cfDNA based assays utilized cutoffs based on dd-cfDNA as a fraction of total recipient serum cfDNA. However, the largest proportion of total recipient cfDNA arises from leukocytes (70–90%) which undergo programmed cell death in the blood, and total cfDNA can increase with conditions such as infection, surgery, and hemodialysis, falsely lowering the calculated dd-cfDNA% [21–24]. To avoid this, multiple prospective studies have demonstrated the utility of the addition of absolute quantification of dd-cfDNA, to enhance the diagnostic accuracy of these assays [5, 25, 26].

Overall, dd-cfDNA based assays have the most robust data for the non-invasive surveillance and diagnosis of acute rejection. Because these assays are serum based, they can be drawn multiple times at prescribed intervals post-transplant to potentially catch early evidence of graft damage that may precede clinically significant allograft dysfunction [19, 27]. This may allow early escalation of immunosuppressive therapy in those patients who demonstrate elevated levels of dd-cfDNA and quicker de-escalation of therapies if levels are low, reducing risk of drug related toxicity and side effects. After treatment for rejection, dd-cfDNA levels return to baseline and can be used to monitor for resolution of rejection [19, 28], or recurrence of rejection, especially critical in patients with AMR [29, 30]. In patients where there is higher suspicion for allograft dysfunction or in patients who present with non-specific symptoms, dd-cfDNA quantification may allow for focusing of diagnostic testing and triage those patients who may most benefit from invasive testing [31].

However, prospective data using dd-cfDNA to guide immunosuppression is lacking. A recent single- center retrospective study looking at heart transplant recipients with a negative biopsy and elevated dd-cfDNA had significantly elevated risk of graft dysfunction and mortality and were associated with changes in immunosuppressive management [32]. However, these were empiric decisions made by the clinicians on a case-by-case basis due to lack of standardized treatment algorithms and outcomes of these strategies remain largely untested.

### Intra-Graft Molecular Profiling

As the use of routine surveillance endomyocardial biopsy (EMB) has waned with the development and widespread use of more robust molecular assays, for-cause EMB remains necessary when rejection is suspected due to clinical signs and symptoms. Traditional evaluation of rejection relies on immunohistologic evaluation of endomyocardial tissue which is limited by significant interobserver variability in both ACR and AMR, cost, and procedural risks [31, 33]. However, when a biopsy is performed, tissue-based gene expression profiling holds promise as a complementary strategy to augment the precision of tissue analysis by evaluating the transcripts associated with tissue injury and rejection with microarrays Molecular Microscope Diagnostic System (MMDx; Transcriptome Sciences). Validation studies in heart transplantation demonstrate distinct gene sets/archetypes that correlated with acute cellular rejection (ACR) and antibody-mediated rejection (AMR) [34]. An earlier study analyzed tissue-based gene expression profiling in patients with AMR, identifying molecular signatures with mRNA transcripts associated with natural killer cell, macrophage, and endothelial cell activation and interferon-gamma activity, and importantly correlated with degree of injury within pAMR ISHLT categories [35]. A more recent intra-graft GEP study revisited the analysis of 1320 EMB samples, finding that many samples previously diagnosed as no rejection actually had a gradient of molecular disease-associated changes with minor increases in transcripts associated with AMR and increasing donor-specific antibody (DSA) positivity [36].

### Peripheral Blood T-Cell Activation

ImmuKnow (Viracor-IBT) is an FDA-approved assay which assesses T-cell immunoreactivity by stimulation of peripheral blood T-cells with phytohemagglutinin and measures ATP production. A retrospective study with 296 patients demonstrated that ATP levels of < 200 ng/ml were associated with infection but assessment of risk of rejection with elevated levels was limited by low number of events [37]. However, subsequent prospect clinical validation did not show that this assay was predictive of acute rejection or clinically significant infection [38]. Currently, the ISHLT gives this a class III recommendation for routine use for rejection monitoring [39].

### Other Emerging Biomarkers (Research Setting Alone)

#### MicroRNAs

MicroRNAs, are small RNA molecules that negatively regulate gene expression and are highly conserved in humans [40]. In endomyocardial tissue samples, microRNAs are altered during ACR and AMR [41, 42]. Multiple studies have demonstrated differential expression in ACR and AMR in blood samples of patients with biopsy proven acute rejection [43–46].

Unlike quantification of dd-cfDNA which may be considered a broad biomarker of allograft injury/rejection, specific groups of microRNAs are dysregulated in ACR and AMR. Therefore, the signatures are unique to each subtype of rejection [44]. Both the innate and adaptive immune systems are regulated by micro RNAs [47]. They are implicated in: bone marrow hematopoietic stem cell response to transforming growth factor-β (TGF-β) via toll-like receptors (TLR) [48], macrophage activation by TLRs and cytokines [49], natural killer (NK) cell activation and inactivation [50], B-cell development and differentiation to plasma cells [51–55], as well T-cell proliferation and differentiation [56–58] Therefore, there has been a strong interest in developing microRNA biomarkers to optimize immunosuppression with two large ongoing multicenter studies in pediatric (OPTIMA, NCT06532890) and adult (OPTIMAL, NCT06939751) heart transplant populations underway.

#### Soluble Protein Markers

Commonly available, cheap serum assays used widely as markers of heart failure and ischemic injury have been studied for their utility in evaluation of suspected graft injury but have shown limited utility in heart transplantation. Specifically, B-type natriuretic peptide (BNP) and N-terminal-pro-B-type natriuretic peptide (NT-proBNP) have not been shown to reliably predict allograft dysfunction or rejection [59–61]. Importantly, however, these studies were limited to ACR. This is a crucial distinction to make as AMR is often associated with higher levels of %dd-cfDNA compared to ACR suggesting more significant graft injury [19] and with higher rates of graft dysfunction and mortality [62].

Additionally, newer generation high sensitivity cardiac troponin T assay have also not been able to reliably diagnose or exclude ACR [63]. Lastly, C-reactive protein and interleukin-6 shown to be non-specific markers of systemic inflammation have not shown to be an accurate diagnostic marker of allograft rejection [64].

#### Proteomics

Proteomics may hold promise in precision evaluation of signaling pathways involved in graft injury, immune response, and inflammation through concurrent analysis of hundreds of protein markers, as specific molecular signatures may suggest type of cell injury. Importantly, the leveraging of machine learning holds promise in addressing the challenge of analyzing the massive amount of data that these studies produce. A recent study from the GRAfT investigators identified specific serum protein expression profiling that could differentiate between AMR and ACR and no rejection, using a sample obtained at a single point in time [65]. In AMR, there was significant upregulation of NT-pro BNP and suppression of tumorigenicity 2 (ST2) levels, known biomarkers of heart failure [66], as well as increases insulin-like growth factor binding protein 2 (IGFBP-2) which had previously been identified in proteomic analysis of kidney transplant patients with graft dysfunction and rejection [67]. In ACR, there was upregulation of inflammatory proteins CXCL10 and CD5 involved in T-cell signaling pathways in cell-mediated graft injury. Proteomic analysis has also been used to identify markers associated with CAV [68, 69] and primary graft dysfunction [70].

Further proteomic studies may aim to develop specific protein signatures that could quantify net state of immunosuppression, characterizing patients as low risk for rejection, under-immunosuppressed and at risk for rejection, or over-immunosuppressed in patients with complications of immunosuppression such as infection or malignancy, allowing for more precise titration of immunosuppressive therapies. Better understanding of these signaling pathways may also provide information on potential therapeutic targets as well.

#### Exosomes

Exosomes are tiny cell-derived lipid-bound extracellular vesicles involved in cell-to-cell communication and they carry dd-cfDNA, mRNA, microRNAs, and proteins. Exosomal proteomic analysis has been studied as a potential novel approach for surveillance and diagnosis of acute transplant rejection. An early study by Kennel et al. analyzed the protein signature of the intra-vesicular contents by mass spectrometry after lysis and identified 15 proteins that were differentially expressed in patients with rejection [71]. A newer study by Burrello et al. examined the proteomics of surface antigens of circulating extra-cellular vesicles analyzed by conventional flow cytometry in patients with histologically diagnosed ACR, with multiple samples collected longitudinally which showed increased surface antigen expression of markers of dendritic cell and T-cell activation during acute episodes of rejection that improved with immunosuppression therapy, reflecting improving clinical trajectory [72]. Importantly, the longitudinal assessment also demonstrated that increased antigen expression preceded EMB identification of ACR.

#### Torque Teno Virus

The quantity of serum cell-free DNA of torque tenovirus (TTV), a highly prevalent and non-pathogenic anellovirus in the human virome has been shown to vary with level of immune competence in kidney and lung transplant patients, and may hold promise in predicting risk of infection and rejection in heart transplant patients [73]. TTV viral load is primarily driven by T-lymphocyte function and counts and varies inversely with conditions that alter T-cell function such as immunosuppression or genetic variation in T-cell regulation [74–76]. Studies have been most robust in kidney transplantation with multiple observational studies demonstrating higher TTV loads in patients with a broad range of infections including opportunistic infections and lower TTV loads in patients with varying types and severity of rejection [77, 78]. As a result, there has been proposed TTV load cut offs for optimal immunosuppression [79]. There are multiple ongoing multi-center trials in kidney transplantation studying precision TTV-guided immunosuppression (TTVguideIT, EUCT number: 2022-500024-30-00, TAOIST, NCT06829719). Studies in heart transplant patients has been limited, with mixed association between viral load and immunosuppression without any demonstrated association with rejection [80, 81]. While TTV load appears to be a promising novel marker to assess immunocompetence in other solid organ transplant populations, further studies are needed to validate its utility in the heart transplantation.

## Immunosuppression Titration

It is well known that cause of death and risk of rejection is dynamic [1] highlighting the need of a shift towards more individualized immunosuppression strategy. Generally, a common immunosuppression strategy is employed despite the complicated nuances of each individual patient’s immune system with variability due to genetic and non-genetic factors including age, organ function, and drug-drug interactions [82]. Changes to medication dosing are often done in a reactive manner, monitoring for drug toxicities and measurement of drug levels within a narrow therapeutic window. As the field of heart transplantation moves towards a more personalized approach to immunosuppression, assessment of graft health should ideally include evaluation of the net state of immunosuppression, to maximize graft function while mitigating the risk of infection and malignancy. The current 2023 International Society for Heart and Lung Transplantation guidelines advocate for the use of noninvasive rejection monitoring modalities including gene expression profiling and dd-cfDNA as part of the surveillance protocol without clear guidance on frequency of testing or the omission of routine EMB if non-invasive tests are reassuring [39]. Ideally, a composite risk prediction tool incorporating pre-transplant risk modifiers such as HLA allosensitization and demographic factors as well as post-transplant assessment, would be utilized longitudinally to allow for a more comprehensive individualized assessment of graft quiescence and overall net state of immunosuppression to guide therapeutic decision making. This would include currently available and commonly used non-invasive modalities such as imaging and DSA assessment as well as newer molecular strategies involving evaluation of dd-cfDNA, microRNA, proteomic analysis and torque teno virus quantification. Further studies are needed to demonstrate the safety and efficacy of multi-omic approach in safely titrating immunosuppression dosing.

## Conclusion

Ideally, a personalized immunosuppression strategy will require integration of multiple data points reflective of the health of the transplanted organ and the immune state. Commercially available dd-cfDNA and GEP assays as well as emerging -omic based modalities hold promise in creating a more personalized assessment of risk and improving the precision of post-transplant care.

## Key References

- Deng MC, Eisen HJ, Mehra MR, Billingham M, Marboe CC, Berry G, et al. Noninvasive discrimination of rejection in cardiac allograft recipients using gene expression profiling. Am J Transplant. 2006;6(1):150-60.
  - ○ This was a landmark multicenter study validating the use of gene expression profiling in detecting absence of moderate to severe acute cellular rejection using an 11 gene real-time PCR assay.
- Snyder TM, Khush KK, Valantine HA, Quake SR. Universal noninvasive detection of solid organ transplant rejection. Proc Natl Acad Sci U S A. 2011;108(15):6229-34.
  - ○ The study demonstrated the use of circulating dd-cfDNA as a biomarker of allograft injury.
- Agbor-Enoh S, Shah P, Tunc I, Hsu S, Russell S, Feller E, et al. Cell-Free DNA to Detect Heart Allograft Acute Rejection. Circulation. 2021;143(12):1184-97.
  - ○ This multicenter, prospective study demonstrated that dd-cfDNA had high sensitivity and specificity in detecting rejection after 28 days post-transplant, often earlier than with endomyocardial biopsy, and may differentiate between antibody-mediated and acute cellular rejection.
- Kim PJ, Olympios M, Sideris K, Tseliou E, Tran TY, Carter S, et al. A two-threshold algorithm using donor-derived cell-free DNA fraction and quantity to detect acute rejection after heart transplantation. Am J Transplant. 2025;25(9):1895-905.
  - ○ The manuscript demonstrated the improved diagnostic accuracy of dd-cfDNA based assays in acute rejection surveillance by addition of absolute dd-cfDNA quantification, in addition to as a fraction of total dd-cfDNA.
- Mehta A, DeFilippis EM, Stehlik J, Jackson AM, Kobashigawa JA, Shah P. Contemporary Antibody-Mediated Rejection in Heart Transplantation: JACC: Heart Failure Position Statement. JACC Heart Fail. 2025;13(10):102614.
  - ○ This position statements highlights the use of novel molecular diagnostics in the contemporary diagnosis of antibody-mediated rejection including dd-cfDNA and intragraft gene expression profiling.

## Data Availability

No datasets were generated or analysed during the current study.

## Declarations

This article does not contain any studies with human or animal subjects performed by any of the authors.

## References

[1] Petroni T, D’Alessandro C, Combes A, Golmard JL, Brechot N, Barreda E, et al. Long-term outcome of heart transplantation performed after ventricular assist device compared with standard heart transplantation. Arch Cardiovasc Dis. 2019;112(8–9):485–93. 10.1016/j.acvd.2019.05.004 31353279

[2] Lund LH, Edwards LB, Kucheryavaya AY, Benden C, Dipchand AI, Goldfarb S, et al. The Registry of the International Society for Heart and Lung Transplantation: Thirty-second Official Adult Heart Transplantation Report–2015; Focus Theme: Early Graft Failure. J Heart Lung Transpl. 2015;34(10):1244–54. 10.1016/j.healun.2015.08.003 26454738

[3] Williams AM, Trahanas JM, Bommareddi S, Lima B, DeVries SA, Lowman J, et al. Rapid Recovery of Donor Hearts for Transplantation after Circulatory Death. N Engl J Med. 2025;393(3):267–74. 10.1056/NEJMoa2500456 40673585

[4] Khush KK, Cherikh WS, Chambers DC, Harhay MO, Hayes D Jr., Hsich E, et al. The International Thoracic Organ Transplant Registry of the International Society for Heart and Lung Transplantation: Thirty-sixth adult heart transplantation report – 2019; focus theme: Donor and recipient size match. J Heart Lung Transpl. 2019;38(10):1056–66. 10.1016/j.healun.2019.08.004 PMC6816343 31548031

[5] Shah P, Kim PJ, Schlendorf KH, Colvin MM, DeVore AD, Eisen HJ, et al. Primary Results of the Development of Non-Invasive Cell-Free DNA to Supplant INvasivE Biopsy in Heart Transplantation (DEFINE-HT) Study. J Heart Lung Transplantation. 2025;44(4):S90.

[6] Hsich E, Singh TP, Cherikh WS, Harhay MO, Hayes D Jr., Perch M, et al. The International thoracic organ transplant registry of the international society for heart and lung transplantation: Thirty-ninth adult heart transplantation report-2022; focus on transplant for restrictive heart disease. J Heart Lung Transpl. 2022;41(10):1366–75. 10.1016/j.healun.2022.07.018 PMC10281817 36031520

[7] Stehlik J, Kobashigawa J, Hunt SA, Reichenspurner H, Kirklin JK. Honoring 50 Years of Clinical Heart Transplantation in Circulation: In-Depth State-of-the-Art Review. Circulation. 2018;137(1):71–87. 10.1161/CIRCULATIONAHA.117.029753 29279339

[8] Multani A, Moayedi Y, Puing A, Henricksen E, Garvert DW, Gomez CA, et al. Recent Trends of Infectious Complications Following Heart Transplantation. Transplantation. 2020;104(10):e284–94. 10.1097/TP.0000000000003307 32413012

[9] Lund LH, Khush KK, Cherikh WS, Goldfarb S, Kucheryavaya AY, Levvey BJ, et al. The Registry of the International Society for Heart and Lung Transplantation: Thirty-fourth Adult Heart Transplantation Report-2017; Focus Theme: Allograft ischemic time. J Heart Lung Transpl. 2017;36(10):1037–46. 10.1016/j.healun.2017.07.019 28779893

[10] Roest S, Hesselink DA, Klimczak-Tomaniak D, Kardys I, Caliskan K, Brugts JJ, et al. Incidence of end-stage renal disease after heart transplantation and effect of its treatment on survival. ESC Heart Fail. 2020;7(2):533–41. 10.1002/ehf2.12585 PMC7160492 32022443

[11] Pinney SP, Balakrishnan R, Dikman S, Nair A, Hammond K, Domanski M, et al. Histopathology of renal failure after heart transplantation: a diverse spectrum. J Heart Lung Transpl. 2012;31(3):233–7. 10.1016/j.healun.2011.08.012 21945725

[12] Youn JC, Stehlik J, Wilk AR, Cherikh W, Kim IC, Park GH, et al. Temporal Trends of De Novo Malignancy Development After Heart Transplantation. J Am Coll Cardiol. 2018;71(1):40–9. 10.1016/j.jacc.2017.10.077 29301626

[13] Ingulli E. Mechanism of cellular rejection in transplantation. Pediatr Nephrol. 2010;25(1):61–74. 10.1007/s00467-008-1020-x PMC2778785 21476231

[14] Deng MC, Eisen HJ, Mehra MR, Billingham M, Marboe CC, Berry G, et al. Noninvasive discrimination of rejection in cardiac allograft recipients using gene expression profiling. Am J Transpl. 2006;6(1):150–60. 10.1111/j.1600-6143.2005.01175.x 16433769

[15] Snyder TM, Khush KK, Valantine HA, Quake SR. Universal noninvasive detection of solid organ transplant rejection. Proc Natl Acad Sci U S A. 2011;108(15):6229–34. 10.1073/pnas.1013924108 PMC3076856 21444804

[16] Grskovic M, Hiller DJ, Eubank LA, Sninsky JJ, Christopherson C, Collins JP, et al. Validation of a Clinical-Grade Assay to Measure Donor-Derived Cell-Free DNA in Solid Organ Transplant Recipients. J Mol Diagn. 2016;18(6):890–902. 10.1016/j.jmoldx.2016.07.003 27727019

[17] Khush KK, Patel J, Pinney S, Kao A, Alharethi R, DePasquale E, et al. Noninvasive detection of graft injury after heart transplant using donor-derived cell-free DNA: A prospective multicenter study. Am J Transpl. 2019;19(10):2889–99. 10.1111/ajt.15339 PMC6790566 30835940

[18] Kim PJ, Olymbios M, Siu A, Wever Pinzon O, Adler E, Liang N, et al. A novel donor-derived cell-free DNA assay for the detection of acute rejection in heart transplantation. J Heart Lung Transpl. 2022;41(7):919–27. 10.1016/j.healun.2022.04.002 PMC9670834 35577713

[19] Agbor-Enoh S, Shah P, Tunc I, Hsu S, Russell S, Feller E, et al. Cell-Free DNA to Detect Heart Allograft Acute Rejection. Circulation. 2021;143(12):1184–97. 10.1161/CIRCULATIONAHA.120.049098 PMC8221834 33435695

[20] Dholakia S, De Vlaminck I, Khush KK. Adding Insult on Injury: Immunogenic Role for Donor-derived Cell-free DNA? Transplantation. 2020;104(11):2266–71. 10.1097/TP.0000000000003240 PMC7590963 32217943

[21] Sun K, Jiang P, Chan KC, Wong J, Cheng YK, Liang RH, et al. Plasma DNA tissue mapping by genome-wide methylation sequencing for noninvasive prenatal, cancer, and transplantation assessments. Proc Natl Acad Sci U S A. 2015;112(40):E5503–12. 10.1073/pnas.1508736112 PMC4603482 26392541

[22] Rhodes A, Wort SJ, Thomas H, Collinson P, Bennett ED. Plasma DNA concentration as a predictor of mortality and sepsis in critically ill patients. Crit Care. 2006;10(2):R60. 10.1186/cc4894 PMC1550922 16613611

[23] Ahmed AI, Soliman RA, Samir S. Cell Free DNA and Procalcitonin as Early Markers of Complications in ICU Patients with Multiple Trauma and Major Surgery. Clin Lab. 2016;62(12):2395–404. 10.7754/Clin.Lab.2016.160615 28164563

[24] Jeong DW, Moon JY, Choi YW, Moon H, Kim K, Lee YH, et al. Effect of blood pressure and glycemic control on the plasma cell-free DNA in hemodialysis patients. Kidney Res Clin Pract. 2015;34(4):201–6. 10.1016/j.krcp.2015.09.002 PMC4688578 26779422

[25] Bohmer J, Wasslavik C, Andersson D, Stahlberg A, Jonsson M, Wahlander H, et al. Absolute Quantification of Donor-Derived Cell-Free DNA in Pediatric and Adult Patients After Heart Transplantation: A Prospective Study. Transpl Int. 2023;36:11260. 10.3389/ti.2023.11260 PMC10641041 37965628

[26] Kim PJ, Olympios M, Sideris K, Tseliou E, Tran TY, Carter S, et al. A two-threshold algorithm using donor-derived cell-free DNA fraction and quantity to detect acute rejection after heart transplantation. Am J Transpl. 2025;25(9):1895–905. 10.1016/j.ajt.2025.04.021 PMC12435761 40334845

[27] Goldberg JF, Tian X, Bon A, Xu Y, Gerhard E, Brower R, et al. Redefining Cardiac Antibody-Mediated Rejection With Donor-Specific Antibodies and Graft Dysfunction. Circ Heart Fail. 2024;17(12):e011592. 10.1161/CIRCHEARTFAILURE.124.011592 PMC12147663 39584219

[28] De Vlaminck I, Valantine HA, Snyder TM, Strehl C, Cohen G, Luikart H, et al. Circulating cell-free DNA enables noninvasive diagnosis of heart transplant rejection. Sci Transl Med. 2014;6(241):241ra77. 10.1126/scitranslmed.3007803 PMC4326260 24944192

[29] Mehta A, DeFilippis EM, Stehlik J, Jackson AM, Kobashigawa JA, Shah P. Contemporary Antibody-Mediated Rejection in Heart Transplantation: JACC: Heart Failure Position Statement. JACC Heart Fail. 2025;13(10):102614. 10.1016/j.jchf.2025.102614 PMC13537435 40848703

[30] Kim PJ, Alam AH, Teuteberg JJ, Khush KK, Pinney SP, Cheng RK, et al. Donor-Derived Cell-Free DNA in Antibody-Mediated Rejection: An Analysis of the Surveillance HeartCare Outcomes Registry. JACC Heart Fail. 2026;14(1):102716. 10.1016/j.jchf.2025.102716 PMC12719789 41123513

[31] Mehta A, Goldberg J, Bagchi P, Marboe C, Shah KB, Najjar SS, et al. Pathologist interrater reliability and clinical implications of elevated donor-derived cell-free DNA beyond heart transplant rejection, on behalf of the GRAfT investigators. J Heart Lung Transplantation. 2025;44(5):803–12. 10.1016/j.healun.2024.10.006 PMC11994287 39424014

[32] Moeller CM, Fernandez Valledor A, Oren D, Rahman S, Baranowska J, Hertz A, et al. Significance of Elevated Donor-Derived Cell-Free DNA in Heart Transplant Recipients With Negative Endomyocardial Biopsies: A Dawn of a New Era. Circ Heart Fail. 2025;18(11):e012787. 10.1161/CIRCHEARTFAILURE.125.012787 41025234

[33] Crespo-Leiro MG, Zuckermann A, Bara C, Mohacsi P, Schulz U, Boyle A, et al. Concordance among pathologists in the second Cardiac Allograft Rejection Gene Expression Observational Study (CARGO II). Transplantation. 2012;94(11):1172–7. 10.1097/TP.0b013e31826e19e2 23222738

[34] Halloran PF, Potena L, Van Huyen JD, Bruneval P, Leone O, Kim DH, et al. Building a tissue-based molecular diagnostic system in heart transplant rejection: The heart Molecular Microscope Diagnostic (MMDx) System. J Heart Lung Transpl. 2017;36(11):1192–200. 10.1016/j.healun.2017.05.029 28662985

[35] Loupy A, Van Duong JP, Hidalgo L, Reeve J, Racape M, Aubert O, et al. Gene Expression Profiling for the Identification and Classification of Antibody-Mediated Heart Rejection. Circulation. 2017;135(10):917–35. 10.1161/CIRCULATIONAHA.116.022907 28148598

[36] Halloran PF, Madill-Thomsen K, Aliabadi-Zuckermann AZ, Cadeiras M, Crespo-Leiro MG, Depasquale EC, et al. Many heart transplant biopsies currently diagnosed as no rejection have mild molecular antibody-mediated rejection-related changes. J Heart Lung Transpl. 2022;41(3):334–44. 10.1016/j.healun.2021.08.004 34548198

[37] Kobashigawa JA, Kiyosaki KK, Patel JK, Kittleson MM, Kubak BM, Davis SN, et al. Benefit of immune monitoring in heart transplant patients using ATP production in activated lymphocytes. J Heart Lung Transpl. 2010;29(5):504–8. 10.1016/j.healun.2009.12.015 20133166

[38] Rossano JW, Denfield SW, Kim JJ, Price JF, Jefferies JL, Decker JA, et al. Assessment of the Cylex ImmuKnow cell function assay in pediatric heart transplant patients. J Heart Lung Transpl. 2009;28(1):26–31. 10.1016/j.healun.2008.10.001 19134527

[39] Velleca A, Shullo MA, Dhital K, Azeka E, Colvin M, DePasquale E, et al. The International Society for Heart and Lung Transplantation (ISHLT) guidelines for the care of heart transplant recipients. J Heart Lung Transpl. 2023;42(5):e1–141. 10.1016/j.healun.2022.10.015 37080658

[40] Shah P, Bristow MR, Port JD. MicroRNAs in Heart Failure, Cardiac Transplantation, and Myocardial Recovery: Biomarkers with Therapeutic Potential. Curr Heart Fail Rep. 2017;14(6):454–64. 10.1007/s11897-017-0362-8 28940102

[41] Van Duong JP, Tible M, Gay A, Guillemain R, Aubert O, Varnous S, et al. MicroRNAs as non-invasive biomarkers of heart transplant rejection. Eur Heart J. 2014;35(45):3194–202. 10.1093/eurheartj/ehu346 25176944

[42] Di Francesco A, Fedrigo M, Santovito D, Natarelli L, Castellani C, De Pascale F, et al. MicroRNA signatures in cardiac biopsies and detection of allograft rejection. J Heart Lung Transpl. 2018;37(11):1329–40. 10.1016/j.healun.2018.06.010 30174164

[43] Constanso-Conde I, Hermida-Prieto M, Barge-Caballero E, Nunez L, Pombo-Otero J, Suarez-Fuentetaja N, et al. Circulating miR-181a-5p as a new biomarker for acute cellular rejection in heart transplantation. J Heart Lung Transpl. 2020;39(10):1100–8. 10.1016/j.healun.2020.05.018 32654912

[44] Shah P, Agbor-Enoh S, Bagchi P, deFilippi CR, Mercado A, Diao G, et al. Circulating microRNAs in cellular and antibody-mediated heart transplant rejection. J Heart Lung Transpl. 2022;41(10):1401–13. 10.1016/j.healun.2022.06.019 PMC9529890 35872109

[45] Sukma Dewi I, Celik S, Karlsson A, Hollander Z, Lam K, McManus JW, et al. Exosomal miR-142-3p is increased during cardiac allograft rejection and augments vascular permeability through down-regulation of endothelial RAB11FIP2 expression. Cardiovasc Res. 2017;113(5):440–52. 10.1093/cvr/cvw244 28073833

[46] Goldberg JF, Bagchi P, Mercado A, Shah KB, Najjar SS, Tchoukina I et al. Identification of Heart Transplant Rejection Subtypes With Circulating MicroRNAs. Circ Heart Fail. 2026:e013141. 10.1161/CIRCHEARTFAILURE.124.013141 PMC12798699 41521923

[47] Mehta A, Baltimore D. MicroRNAs as regulatory elements in immune system logic. Nat Rev Immunol. 2016;16(5):279–94. 10.1038/nri.2016.40 27121651

[48] Taganov KD, Boldin MP, Chang KJ, Baltimore D. NF-kappaB-dependent induction of microRNA miR-146, an inhibitor targeted to signaling proteins of innate immune responses. Proc Natl Acad Sci USA. 2006;103(33):12481–6. 10.1073/pnas.0605298103 PMC1567904 16885212

[49] O’Connell RM, Taganov KD, Boldin MP, Cheng G, Baltimore D. MicroRNA-155 is induced during the macrophage inflammatory response. Proc Natl Acad Sci USA. 2007;104(5):1604–9. 10.1073/pnas.0610731104 PMC1780072 17242365

[50] Beaulieu AM, Bezman NA, Lee JE, Matloubian M, Sun JC, Lanier LL. MicroRNA function in NK-cell biology. Immunol Rev. 2013;253(1):40–52. 10.1111/imr.12045 PMC3621029 23550637

[51] Chen CZ, Li L, Lodish HF, Bartel DP. MicroRNAs modulate hematopoietic lineage differentiation. Science. 2004;303(5654):83–6. 10.1126/science.1091903 14657504

[52] McHeyzer-Williams M, Okitsu S, Wang N, McHeyzer-Williams L. Molecular programming of B cell memory. Nat Rev Immunol. 2011;12(1):24–34. 10.1038/nri3128 PMC3947622 22158414

[53] Barnes NA, Stephenson S, Cocco M, Tooze RM, Doody GM. BLIMP-1 and STAT3 counterregulate microRNA-21 during plasma cell differentiation. J Immunol. 2012;189(1):253–60. 10.4049/jimmunol.1101563 22634616

[54] Lu D, Nakagawa R, Lazzaro S, Staudacher P, Abreu-Goodger C, Henley T, et al. The miR-155-PU.1 axis acts on Pax5 to enable efficient terminal B cell differentiation. J Exp Med. 2014;211(11):2183–98. 10.1084/jem.20140338 PMC4203942 25288398

[55] Porstner M, Winkelmann R, Daum P, Schmid J, Pracht K, Corte-Real J, et al. miR-148a promotes plasma cell differentiation and targets the germinal center transcription factors Mitf and Bach2. Eur J Immunol. 2015;45(4):1206–15. 10.1002/eji.201444637 25678371

[56] Murugaiyan G, da Cunha AP, Ajay AK, Joller N, Garo LP, Kumaradevan S, et al. MicroRNA-21 promotes Th17 differentiation and mediates experimental autoimmune encephalomyelitis. J Clin Investig. 2015;125(3):1069–80. 10.1172/JCI74347 PMC4362225 25642768

[57] Wu H, Neilson JR, Kumar P, Manocha M, Shankar P, Sharp PA, et al. miRNA profiling of naive, effector and memory CD8 T cells. PLoS ONE. 2007;2(10):e1020. 10.1371/journal.pone.0001020 PMC2000354 17925868

[58] Yao R, Ma YL, Liang W, Li HH, Ma ZJ, Yu X, et al. MicroRNA-155 modulates Treg and Th17 cells differentiation and Th17 cell function by targeting SOCS1. PLoS ONE. 2012;7(10):e46082. 10.1371/journal.pone.0046082 PMC3473054 23091595

[59] Arnau-Vives MA, Almenar L, Hervas I, Osa A, Martinez-Dolz L, Rueda J, et al. Predictive value of brain natriuretic peptide in the diagnosis of heart transplant rejection. J Heart Lung Transpl. 2004;23(7):850–6. 10.1016/j.healun.2003.08.005 15261180

[60] Battes LC, Caliskan K, Rizopoulos D, Constantinescu AA, Robertus JL, Akkerhuis M, et al. Repeated measurements of NT-pro-B-type natriuretic peptide, troponin T or C-reactive protein do not predict future allograft rejection in heart transplant recipients. Transplantation. 2015;99(3):580–5. 10.1097/TP.0000000000000378 25136844

[61] Hammerer-Lercher A, Mair J, Antretter H, Ruttmann E, Poelzl G, Laufer G, et al. B-type natriuretic peptide as a marker of allograft rejection after heart transplantation. J Heart Lung Transpl. 2005;24(9):1444. 10.1016/j.healun.2004.08.018 16143273

[62] Cole RT, Gandhi J, Bray RA, Gebel HM, Yin M, Shekiladze N, et al. Racial differences in the development of de-novo donor-specific antibodies and treated antibody-mediated rejection after heart transplantation. J Heart Lung Transpl. 2018;37(4):503–12. 10.1016/j.healun.2017.11.003 29198929

[63] Fitzsimons SJ, Evans JDW, Rassl DM, Lee KK, Strachan FE, Parameshwar J, et al. High-sensitivity Cardiac Troponin Is Not Associated With Acute Cellular Rejection After Heart Transplantation. Transplantation. 2022;106(5):1024–30. 10.1097/TP.0000000000003876 34241986

[64] Eisenberg MS, Chen HJ, Warshofsky MK, Sciacca RR, Wasserman HS, Schwartz A, et al. Elevated levels of plasma C-reactive protein are associated with decreased graft survival in cardiac transplant recipients. Circulation. 2000;102(17):2100–4. 10.1161/01.cir.102.17.2100 11044427

[65] Goldberg JF, deFilippi CR, Lockhart C, McNair ER, Sinha SS, Kong H, et al. Proteomics in Acute Heart Transplant Rejection, on Behalf of the GRAfT Investigators. Transplantation. 2025;109(7):1230–40. 10.1097/TP.0000000000005258 PMC12130342 39630098

[66] de Lange I, Petersen TB, de Bakker M, Akkerhuis KM, Brugts JJ, Caliskan K, et al. Heart failure subphenotypes based on repeated biomarker measurements are associated with clinical characteristics and adverse events (Bio-SHiFT study). Int J Cardiol. 2022;364:77–84. 10.1016/j.ijcard.2022.06.020 35714717

[67] Wang C, Feng G, Zhao J, Xu Y, Li Y, Wang L, et al. Screening of novel biomarkers for acute kidney transplant rejection using DIA-MS based proteomics. Proteom Clin Appl. 2024;18(3):e2300047. 10.1002/prca.202300047 38215274

[68] Dalsgaard RG, Clemmensen TS, Eiskjaer H, Poulsen SH, Bjerre KP. Proteomics early after heart transplantation and relation to coronary intimal changes and prognosis. JHLT Open. 2024;5:100110. 10.1016/j.jhlto.2024.100110 PMC11935488 40143911

[69] Almufleh A, Zhang L, Mielniczuk LM, Stadnick E, Davies RA, Du Q, et al. Biomarker discovery in cardiac allograft vasculopathy using targeted aptamer proteomics. Clin Transpl. 2020;34(1):e13765. 10.1111/ctr.13765 31815308

[70] Truby LK, Kwee LC, Agarwal R, Grass E, DeVore AD, Patel CB, et al. Proteomic profiling identifies CLEC4C expression as a novel biomarker of primary graft dysfunction after heart transplantation. J Heart Lung Transpl. 2021;40(12):1589–98. 10.1016/j.healun.2021.07.024 PMC8670564 34511330

[71] Kennel PJ, Saha A, Maldonado DA, Givens R, Brunjes DL, Castillero E, et al. Serum exosomal protein profiling for the non-invasive detection of cardiac allograft rejection. J Heart Lung Transpl. 2018;37(3):409–17. 10.1016/j.healun.2017.07.012 28789823

[72] Burrello J, Panella S, Barison I, Castellani C, Burrello A, Airale L, et al. Machine learning-assisted assessment of extracellular vesicles can monitor cellular rejection after heart transplant. Commun Med (Lond). 2025;5(1):288. 10.1038/s43856-025-00999-0 PMC12246144 40640467

[73] De Vlaminck I, Khush KK, Strehl C, Kohli B, Luikart H, Neff NF, et al. Temporal response of the human virome to immunosuppression and antiviral therapy. Cell. 2013;155(5):1178–87. 10.1016/j.cell.2013.10.034 PMC4098717 24267896

[74] Focosi D, Macera L, Boggi U, Nelli LC, Maggi F. Short-term kinetics of torque teno virus viraemia after induction immunosuppression confirm T lymphocytes as the main replication-competent cells. J Gen Virol. 2015;96(Pt 1):115–7. 10.1099/vir.0.070094-0 25304651

[75] Ramzi M, Iravani Saadi M, Zarei T, Yaghobi R, Arandi N. Association Between Cytotoxic T-Lymphocyte Antigen 4 Gene Polymorphisms and Torque Teno Virus Infection After Hematopoietic Stem Cell Transplantation. Exp Clin Transpl. 2021;19(3):259–63. 10.6002/ect.2017.0105 29251582

[76] Maggi F, Ricci V, Bendinelli M, Nelli LC, Focosi D, Papineschi F, et al. Changes In CD8 + 57+ T lymphocyte expansions after autologous hematopoietic stem cell transplantation correlate with changes in torquetenovirus viremia. Transplantation. 2008;85(12):1867–8. 10.1097/TP.0b013e31817615e6 18580484

[77] Doberer K, Schiemann M, Strassl R, Haupenthal F, Dermuth F, Gorzer I, et al. Torque teno virus for risk stratification of graft rejection and infection in kidney transplant recipients-A prospective observational trial. Am J Transpl. 2020;20(8):2081–90. 10.1111/ajt.15810 PMC7496119 32034850

[78] Fernandez-Ruiz M, Albert E, Gimenez E, Ruiz-Merlo T, Parra P, Lopez-Medrano F, et al. Monitoring of alphatorquevirus DNA levels for the prediction of immunosuppression-related complications after kidney transplantation. Am J Transpl. 2019;19(4):1139–49. 10.1111/ajt.15145 30346659

[79] Doberer K, Kapps S, Haupenthal F, Bond G. Immune Monitoring Goes Viral - Torque Teno Virus for Immunologic Risk Stratification After Kidney Transplantation. Transpl Int. 2025;38:15074. 10.3389/ti.2025.15074 PMC12660151 41323957

[80] Uyanik-Uenal K, Perkmann-Nagele N, Wittmann F, Ceran E, Aliabadi-Zuckermann A, Laufer G, et al. Torque Teno Virus DNA Load after Heart Transplantation and Its Association with the Strength of Immunosuppression: Preliminary Data of a Prospective Single Center Study. J Heart Lung Transplantation. 2020;39(4):S128.

[81] Berg R, Clemmensen TS, Petersen MS, Mogensen LJH, Christiansen M, Rolid K, et al. Kinetics of Torque Teno virus in heart transplant patients. Hum Immunol. 2023;84(12):110720. 10.1016/j.humimm.2023.110720 37867096

[82] Thervet E, Anglicheau D, Legendre C, Beaune P. Role of pharmacogenetics of immunosuppressive drugs in organ transplantation. Ther Drug Monit. 2008;30(2):143–50. 10.1097/FTD.0b013e31816babef 18367973

